# Development and Characterization of Azithromycin-Loaded Microemulsions: A Promising Tool for the Treatment of Bacterial Skin Infections

**DOI:** 10.3390/antibiotics11081040

**Published:** 2022-08-02

**Authors:** Angela Abruzzo, Carola Parolin, Martina Rossi, Beatrice Vitali, Concettina Cappadone, Federica Bigucci

**Affiliations:** Department of Pharmacy and Biotechnology, University of Bologna, Via San Donato 19/2, 40127 Bologna, Italy; carola.parolin@unibo.it (C.P.); martina.rossi12@unibo.it (M.R.); b.vitali@unibo.it (B.V.); concettina.cappadone@unibo.it (C.C.); federica.bigucci@unibo.it (F.B.)

**Keywords:** microemulsions, azithromycin, skin infections, permeation/retention studies, topical delivery, methicillin-resistant *Staphylococcus aureus*

## Abstract

In recent years, the treatment of bacterial skin infections has been considered a major healthcare issue due to the growing emergence of antibiotic-resistant strains of *Staphylococcus aureus*. The incorporation of antibiotics in appropriate nanosystems could represent a promising strategy, able to overcome several drawbacks of the topical treatment of infections, including poor drug retention within the skin. The present work aims to develop microemulsions containing azithromycin (AZT), a broad-spectrum macrolide antibiotic. Firstly, AZT solubility in various oils, surfactants and co-surfactants was assessed to select the main components. Subsequently, microemulsions composed of vitamin E acetate, Labrasol^®^ and Transcutol^®^ P were prepared and characterized for their pH, viscosity, droplet size, zeta potential and ability to release the drug and to promote its retention inside porcine skin. Antimicrobial activity against *S. aureus* methicillin-resistant strains (MRSA) and the biocompatibility of microemulsions were evaluated. Microemulsions showed an acceptable pH and were characterized by different droplet sizes and viscosities depending on their composition. Interestingly, they provided a prolonged release of AZT and promoted its accumulation inside the skin. Finally, microemulsions retained AZT efficacy on MRSA and were not cytotoxic. Hence, the developed AZT-loaded microemulsions could be considered as useful nanocarriers for the treatment of antibiotic-resistant infections of the skin.

## 1. Introduction

Bacterial skin infections represent one of the major healthcare issues affecting people worldwide, often determining frequent emergency recovering [1]. They are usually caused by pathogenic bacteria that invade the epidermis and the dermis and can be distinguished in superficially localized or deeper skin infections. Traditionally, *Staphylococcus aureus*, which is part of the normal skin flora in approximately 30% of people, has been classified as the leading cause of skin infections [2]. Since 1980, antibiotic-resistant *S. aureus* strains have risen and spread, determining the insurgence of infections that are difficult to eradicate and representing a public health problem. Indeed, nowadays, multi-drug resistant (MDR) and methicillin-resistant (MRSA) *S. aureus* are a major cause for nosocomial and community infections throughout the world [3]. In particular, skin infections caused by antibiotic-resistant *S. aureus* strains have been considered hard-to-treat and more and more measures are urgently needed to control and overcome antibiotic resistance [4]. Among these, it is worth mentioning the identification of new antibiotic molecules or the combination of currently approved antibiotics with nanotechnology. The latter is considered as one of the most relevant approaches, also taking into account the long process needed for the development of new drugs. In this context, the design of new nanosystems useful for the treatment of antibiotic-resistant bacterial infections of the skin still remains a challenge of growing urgency.

The topical application of antibiotics is generally adopted as the first strategy for the treatment of skin infections. However, the poor drug retention within the skin can lead to an insufficient concentration of the active molecule at the action site, which consequently requires multiple and frequent administrations. In order to improve antibiotic delivery, the employment of nanosystems, such as emulsions-based nanosystems (nanoemulsions and microemulsions), polymeric nanoparticles and liposome-related nanosystems (liposomes, transferosomes and ethosomes), represents a promising approach. Indeed, it has been reported that nanosystems are potentially able to improve the efficacy of antibiotics and to reduce the risk of their overuse, which is responsible for antimicrobial resistance [5,6,7]. This is strictly linked to the peculiar properties of nanosystems, such as their restricted dimension, high superficial area and high reactivity to pathogen microorganisms [8].

Nowadays, microemulsions (MEs), monophasic optically isotropic and transparent formulations, are mostly proposed for their capability to enhance drug uptake into the skin [9,10]. Differently from the previously mentioned nanosystems (polymeric nanoparticles, liposome related nanosystems and nanoemulsions), MEs are thermodynamically stable and can be formed without any significant energy input and organic solvents by mixing the oily phase and the aqueous phase in the presence of amphiphilic substances and co-surfactants, which is able to decrease the interfacial tension to a very low value. Besides their ability to promote drug accumulation within the skin, other advantages include the spontaneous and simple formation and consequent ease of industrial scale-up, the reduced droplet size, the high solubilization potential, especially for poorly soluble drugs, and the prolonged drug release [11,12]. To date, different antibiotic-loaded microemulsions have been designed for the treatment of skin infections. Peira and co-workers [13] developed microemulsions containing azelaic acid for the treatment of acne and found that microemulsions facilitated azelaic acid diffusion through the skin. In another study, microemulsions loaded with cephalexin monohydrate were developed for the treatment of skin infections; the authors stated that microemulsions maintained the antimicrobial activity of the drug [14]. More recently, ciprofloxacin-loaded microemulsions were designed by Volpe and co-workers; the developed formulations presented the ability to disrupt *S. aureus* biofilms and could be proposed for the treatment of skin infections [15].

In this study, azithromycin (AZT) was selected among the various antibiotics. AZT is a broad-spectrum antibiotic, belonging to the macrolide group, able to bind the 50S ribosomal subunit of bacteria, thereby inhibiting the vital process of protein synthesis [16]. Until now, in the field of skin delivery, AZT has been delivered by liposomes or zinc oxide nanoparticles for the treatment of topical infections [17] and infected wounds [18], respectively. However, one of the major problems with this drug is the poor solubility [19], which is expected to increase by using suitable delivery systems, such as microemulsions. So far, AZT-loaded microemulsions have been developed by Fanun and Salim and colleaugues [20,21]. In the first study, the effect of the water fraction on the type of AZT-loaded microemulsion was evaluated. In the second one, the authors developed microemulsions as an ocular system for the delivery of AZT, and the influence of the components on the drug release and permeability through rabbit cornea was investigated. 

In this work, we developed for the first time AZT-loaded microemulsions as innovative nanocarriers in order to obtain an advanced and improved treatment of bacterial skin infections. Specifically, the aim of this study was to combine the advantages of microemulsions, such as the simple and economical preparation, the long-term stability and the high solubility degree of poorly soluble drugs, with their ability to control the drug release and to enhance its retention inside the skin, by exploiting their composition, physico-chemical and functional properties. Our hypothesis was that an increased AZT accumulation inside the skin can allow us to maximize the therapeutic effect and to optimize the efficacy of the treatment of skin infections by reducing the administration frequency and limiting the risk of drug overuse that is generally responsible of antimicrobial resistance. Briefly, the main steps of this study were: (a) to select microemulsion excipients able to enhance AZT solubility; (b) to prepare microemulsions and to characterize them in terms of pH, viscosity, droplet size, polydispersity index, zeta-potential and stability; (c) to investigate the microemulsion’s ability to release the drug and to promote its accumulation within porcine skin; (d) to evaluate the antimicrobial activity of AZT-loaded microemulsions against MRSA strains; (e) to test microemulsion biocompatibility. 

## 2. Results and Discussion

### 2.1. Solubility of AZT

Due to its low solubility in water, it is of great importance to screen those excipients for the preparation of microemulsions in which AZT can be easily dissolved [22].

**Figure 1:**
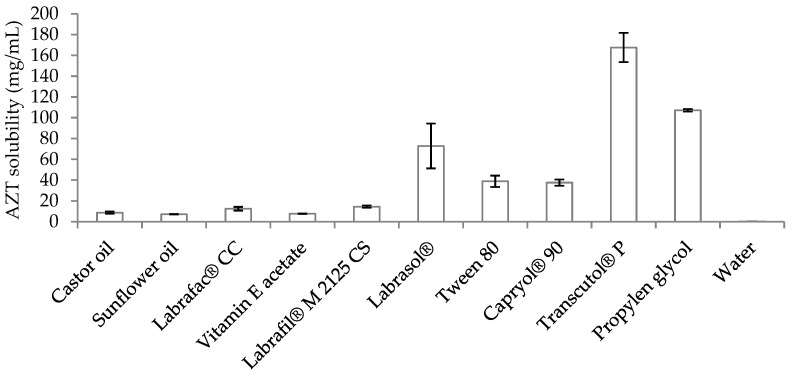
Solubility (mg/mL) of AZT in various types of oils, surfactants and co-surfactants at +25 °C. Data are expressed as means ± SD, n = 3.

Therefore, in order to choose the appropriate components, the solubility of AZT in various oils (castor oil, sunflower oil, Labrafac^®^ CC and vitamin E acetate), surfactants (Labrafil^®^ M 2125 CS, Labrasol^®^, Capryol^®^ 90 and Tween 80) and co-surfactants (Transcutol^®^ P and propylene glycol) was measured. As shown in Figure 1, for all of the tested components, the value of drug solubility was dramatically improved compared to that in water, which resulted in being equal to 0.14 ± 0.02 mg/mL (in agreement with previous results) [19]. Among the tested oils, the highest AZT solubility was reached in Labrafac^®^ CC (12.45 ± 1.80 mg/mL) and the lowest in sunflower oil (7.08 ± 0.21 mg/mL), while no significant difference (*p* > 0.05) was observed between castor oil (8.63 ± 1.04 mg/mL) and vitamin E acetate (7.62 ± 0.21 mg/mL). However, the final selection among the tested oils was conducted depending on the emulsification efficiency; in fact, when Labrafac^®^ CC was employed in association with the other selected components, only coarse macroemulsions were obtained. Among the remaining oils, vitamin E acetate was selected and tested as the oil phase for microemulsion preparation. The choice of vitamin E acetate was made also considering its favorable properties, such as the highly acceptable profile and the well-known topical beneficial effects [23]. Additionally, in recent years, different studies have proposed the employment of vitamin E acetate as edible oil for the development of oil-in-water nanoemulsions [24,25], thus further confirming its greater potentiality. Regarding AZT solubility in the different employed surfactants and co-surfactants, the highest solubilization of the drug was achieved in Labrasol^®^ (77.78 ± 21.55 mg/mL) and Transcutol^®^ P (167.53 ± 14.07 mg/mL), which were selected for the next studies. Labrasol^®^ is one of the commercially available surfactants, also approved for oral administration [26], and has a low toxicity level as a non-ionic surfactant [27]. Transcutol^®^ P is an amphiphilic liquid with a proven ability to increase the dermal and transdermal transport of drugs and their solubility [28]. 

The employment of vitamin E acetate, Labrasol^®^ and Transcutol^®^ P as microemulsion components can ensure an easy AZT solubilization, which is required for microemulsion preparation.

### 2.2. Pseudoternary Phase Diagrams

**Figure 2:**
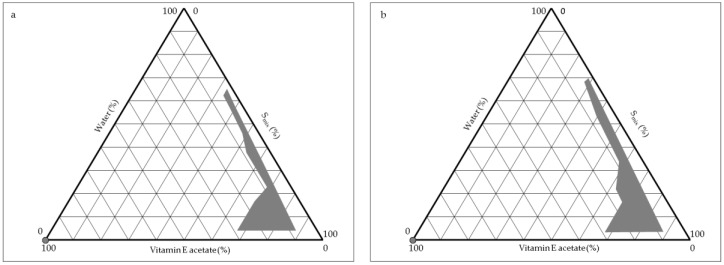
Pseudoternary phase diagrams of the systems composed of oil, surfactant/co-surfactant (S_mix_) and water. The oil phase is vitamin E acetate; S_mix_ is a mixture of Labrasol^®^/Transcutol^®^ P 2:1 *v*/*v* (**a**) and 3:1 *v*/*v* (**b**). The grey regions indicate transparent and homogenous microemulsions; in the remaining regions, coarse turbid emulsions or phase separated systems were observed.

Pseudoternary phase diagrams were built to establish the area with the greatest amplitude for the microemulsion formation (Figure 2). The highlighted grey area within the diagram represents the transparent microemulsion region containing various percentages of components, whereas the remaining area represents the turbid and simple emulsions based on visual observation. Various proportions between the amounts of Labrasol^®^ and Transcutol^®^ P were considered (S_mix_ of 1:1, 2:1, 3:1 *v*/*v*). When S_mix_ 1:1 *v*/*v* was employed, microemulsions were obtained only with ratios of oil/S_mix_ equal to 1/9 *v*/*v* and 1.25/8.75 *v*/*v*, and with a water content of only 5% *w*/*w* (data not shown). Instead, microemulsion regions with a greater area were obtained by using S_mix_ 2:1 *v*/*v* and 3:1 *v*/*v* (regions highlighted in grey in the Figure 2a,b). The results revealed that the maximum area for the formation of microemulsions occurs with S_mix_ 3:1 *v*/*v*. This observation was consistent with previous reports stating that, as the surfactant was increased in the S_mix_ ratio, a higher microemulsion region was observed due to the reduction in the interfacial tension, which enhanced the entropy of the system [29,30]. For S_mix_ 2:1 *v*/*v*, slightly opalescent formulations were obtained for oil/S_mix_ equal to 1/9 *v*/*v* with a water content higher than 67% *w*/*w*, for oil/S_mix_ equal to 1.25/8.75 *v*/*v* with a water content higher than 40% *w*/*w* and for oil/S_mix_ equal to 2/8 *v*/*v* with a water content higher than 20% *w*/*w*. For S_mix_ 3:1 *v*/*v*, slightly opalescent formulations were obtained for oil/S_mix_ equal to 1/9 *v*/*v* with a water content higher than 78% *w*/*w*, for oil/S_mix_ equal to 1.25/8.75 *v*/*v* with a water content higher than 56% *w*/*w* and for oil/S_mix_ equal to 2/8 *v*/*v* with a water content higher than 32% *w*/*w*. These results allowed us to investigate the effect of different surfactant/co-surfactant ratios on the extent of the stable microemulsion region and to select the concentrations of components for microemulsion preparation.

### 2.3. Selection of Microemulsions

#### 2.3.1. Selection and Preparation of Unloaded Microemulsions

Based on the results shown in Figure 2a,b, microemulsions M1, M2 and M3, whose compositions are reported in Table 1, were selected for further experiments. These formulations differ in terms of oil content (7.7% *w*/*w*- for M1 and M2 and 5.9% *w*/*w-* for M3), and S_mix_ (2:1 *v*/*v* for M1 and 3:1 *v*/*v* for M2 and M3). Furthermore, these three formulations were those that satisfied a higher oil:S_mix_ ratio (1.25:8.75 *v*/*v*) and a low content of surfactant (lower than 45% *w*/*w*). In fact, it is noteworthy to limit the amount of surfactant being used, as the greater the amount of surfactant in microemulsions, the greater the risk of developing skin irritation [31]. 

In order to determine the microemulsion type, the electrical conductivity of the selected formulations was measured. The conductivity of the microemulsions M1, M2 and M3 increased with the increase in water content and, specifically, the values were 16.12 ± 0.13 µS/cm, 15.24 ± 0.03 µS/cm and 24.2 ± 0.99 µS/cm, respectively. In accordance with previously mentioned classification [32], all of the developed formulations were oil-in-water microemulsions.

The selected microemulsions were spontaneously formed at room temperature through a simple method only consisting of mixing the different components without specialized equipment, such as mixers and homogenizers. This spontaneous and simple production method is of great value, especially in the case of large-scale production.

#### 2.3.2. Content Determination and Preparation of Loaded Microemulsions

The evaluation of the drug content inside the microemulsions represents a key factor in order to estimate the drug amount in the solubilized form [22], able to be easily released and to be retained inside the skin. The analysis regarding the maximum loading content of AZT in formulations demonstrated that drug solubility in microemulsions was greatly improved compared to that in an aqueous solution (0.14 ± 0.02 mg/mL). Additionally, the AZT solubility varied according to the microemulsion composition and, for M1, M2 and M3, the drug solubility values were equal to 22.09 ± 0.15 mg/mL, 25.02 ± 0.32 mg/mL and 15.10 ± 0.10 mg/mL, respectively. This result could be mainly attributed to the presence of different amounts of the surfactant in the formulations. Generally, it was observed that the presence of an increasing amount of surfactant determined an improved drug solubilization [33]. Moreover, the relative solubility of the drug in various components could contribute to the drug solubility in the final microemulsions.

**Figure 3:**
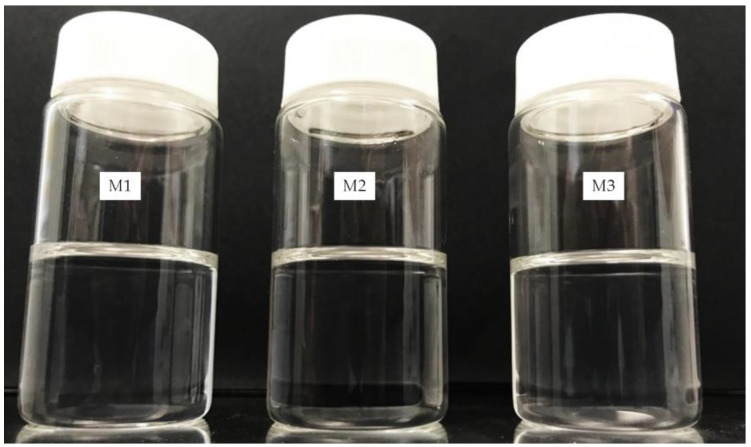
Appearance of loaded microemulsions M1, M2 and M3.

For a more rational comparison among the three formulations, a fixed concentration of 10 mg/mL of AZT was used for the preparation of loaded microemulsions. The obtained loaded microemulsions were homogeneous and transparent (Figure 3).

### 2.4. Characterization of Unloaded and AZT-Loaded Microemulsions

The selected microemulsions were subjected to further characterization in terms of pH, viscosity, mean droplet size, PDI and zeta potential.

#### 2.4.1. Determination of pH and Viscosity

The determination of the pH and viscosity of microemulsions was performed to evaluate the suitability of formulations for topical application. Loaded microemulsions were found to have pH values of around 4.5 (no significant difference was observed between loaded and unloaded microemulsions; *p* > 0.05), which is in the acceptable and tolerable pH range of the skin (i.e., 4.5–5.5), so they would not be a determinant factor of potential skin irritation after their application on the skin [34]. Moreover, loaded M1, M2 and M3 were found to exhibit viscosity values equal to 31.68 ± 0.15 mPa∙s, 36.39 ± 0.17 mPa∙s and 23.99 ± 0.18 mPa∙s (no significant difference was observed between loaded and unloaded microemulsions; *p* > 0.05). This result could be related to the different water content of M1, M2 and M3. In fact, it has been reported that water reduces the interaction between hydrophilic head-groups of the surfactant and thus decreases the viscosity [35]. Indeed, microemulsion M3, which possessed the highest water content (i.e., 48.8% *w*/*w*), showed the lowest viscosity value. In addition, a direct relationship between the surfactant-to-co-surfactant weight ratio and the formulation viscosity was found. In fact, although the water contents of M1 and M2 were similar, M2 exhibited a higher viscosity compared with M1 (*p* < 0.05), which can be attributed to its higher surfactant-to-co-surfactant ratio (S_mix_ 2:1 *v*/*v* for M1 and S_mix_ 3:1 *v*/*v* for M2) [36]. The obtained values of viscosity could impact the drug release behavior, since it has been reported that formulations characterized by low viscosity allow for an easy drug diffusion through the vehicle [37]. Although an increase in viscosity is often desired for liquid microemulsions intended for skin application, a low viscosity could have some beneficial characteristics, such as an easier administration via spray systems.

#### 2.4.2. Mean Droplet Size, Size Distribution and Zeta Potential Measurements

Microemulsion characterization in terms of size, PDI and zeta potential is an important subject to be considered during the development steps, especially for a formulation intended for skin application [38]. Specifically, the droplet size can influence the functional properties of the microemulsions, such as the drug release and its accumulation inside the skin. Microemulsions with a low droplet size (large surface area/volume ratio) may favor the release of the drug and promote its transport into or across the skin [39,40]. According to Table 2, the formulations possessed a droplet size in the range of 94–138 nm, which varied accordingly to the microemulsion composition. In particular, among all of the microemulsions, M1 showed the largest mean droplet size due to its high cosurfactant content, which, in turn, affects the property of the surfactant curvature. In fact, it has been reported that the presence of greater amounts of co-surfactant causes a higher expansion of the surfactant-based film [41]. However, despite being composed of a higher amount of co-surfactant with respect to M3, the M2 droplet size was lower than M3 (*p* < 0.05), probably due to its high content of surfactant, which contributed to lowering the interfacial tension, leading to a decrease in droplet size [42]. Moreover, in the case of loaded microemulsions, an increase in droplet size was observed with respect to unloaded formulations (*p* < 0.05), probably due to the incorporation of AZT inside the droplet, which determined an increase in their size. Regarding PDI, all formulations were found to exhibit PDI values below 0.3, which indicates a narrow distribution of the mean droplet size [43].

The determination of the zeta potential of microemulsions is a crucial factor considering that it can influence the accumulation of the drug inside the skin, as well as the stability of the system. All tested formulations showed negative zeta potential values ranging from −22.32 to −32.60 mV. The negative charge of the prepared microemulsions could be attributed to the anionic groups of the fatty acids and glycols present in the oil, surfactant and co-surfactant [44]. Generally, negatively surface-charged nanocarriers were considered to be more effective than positive or neutral ones for dermal drug delivery. In fact, it has been reported that negatively charged nanocarriers generally improve drug accumulation in the skin [45]. This is also in agreement with data reported by Carrer and co-workers, who demonstrated that negative charged liposomes may allow for a better efficiency of drug penetration within the skin [46]. Moreover, zeta potential values have a role in microemulsion stabilization because the presence of a high electrical charge in the system causes repulsion between droplets, leading to limited aggregation [47]. Finally, loaded microemulsions displayed a lower negative zeta potential with respect to the unloaded ones (*p* < 0.05). This result could be related to the presence of positive charges of AZT (pKa 8.5) [18,48,49] at the pH conditions of the microemulsions, which could contribute to reducing the negative charges of the microemulsions.

### 2.5. Stability

**Figure 4:**
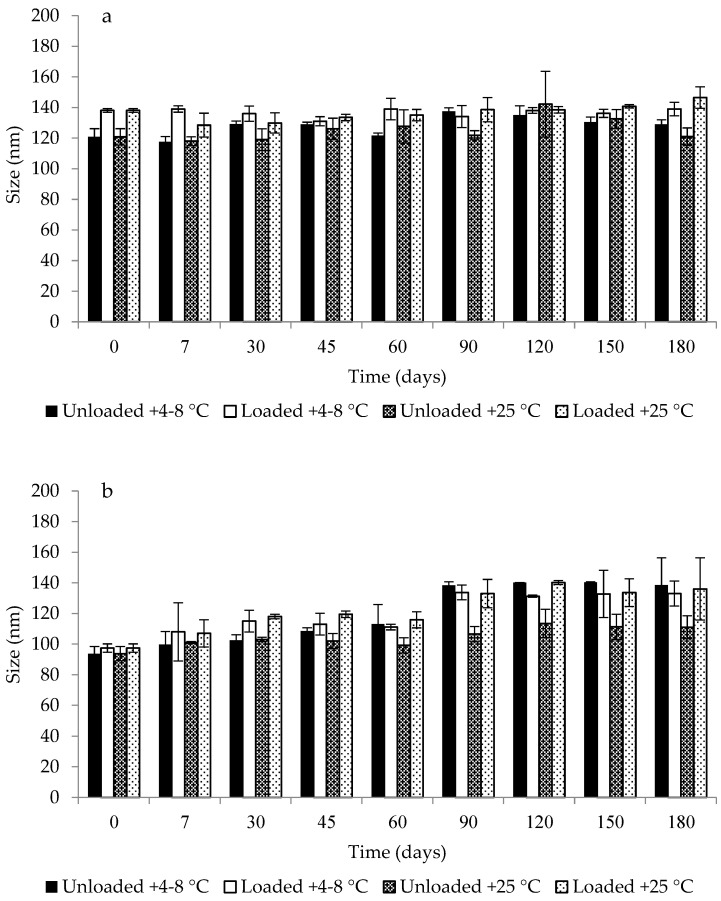

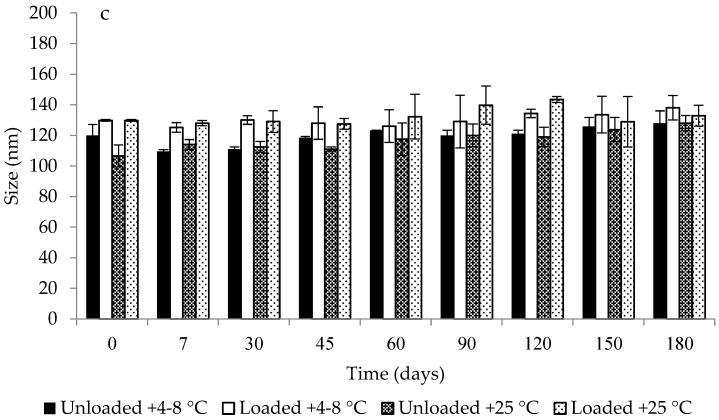
Size variation in unloaded and loaded microemulsions M1 (**a**), M2 (**b**) and M3 (**c**) during 180 days of storage at +4–8 °C and +25 °C. Data are expressed as means ± SD, n = 3.

Stability studies were carried out to ensure that developed microemulsions retained their suitability for use after the storage period. No phase separation or color change was observed during the storage period. In the case of heating–cooling cycles and freeze–thaw cycles, the selected microemulsions demonstrated reversible a turbidity that easily disappeared by being restored at room temperature, in agreement with previous results [49]. Moreover, the mean droplet size and PDI of the selected unloaded and loaded microemulsions were monitored over a storage period of 180 days at +4–8 °C and +25 °C. According to the stability results presented in Figure 4, the selected formulations showed a slight increase in droplet size after 180 days, which was significantly different only for M2 (*p* < 0.05). However, the observed variation can be considered irrelevant with respect to t = 0, since the droplet size remained within the range of 20 to 200 nm, which is the range in which these systems could be defined as microemulsions [50]. The stability of microemulsions could be ascribed to their zeta potential values, which, as previously described, contributed to determining the repulsion between droplets and, consequently, to reducing the aggregation phenomenon [47]. Considering these results, microemulsions can be stored for 180 days at both tested temperatures. Differently from conventional emulsions or other nanocarriers (such as polymeric nanoparticles or liposomes-related nanosystems), microemulsions are stable due to their thermodynamic nature. This result is of great relevance when also taking into account a possible production at industrial scale.

### 2.6. In Vitro Release Studies

**Figure 5:**
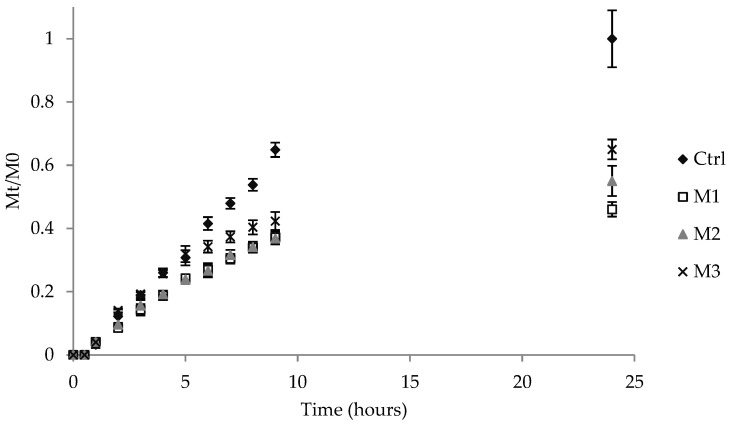
Cumulative drug amount (expressed as fractional amount Mt/M0, where Mt represents the amount of AZT released at each time and M0 the total AZT mass) released from microemulsions and control (Ctrl) plotted as a function of time. Data are expressed as means ± SD, n = 3.

Figure 4 shows the in vitro release profiles of AZT from the control solution and the different microemulsions. Generally, drug release from microemulsions depends on various parameters, such as the oil-to-aqueous-phase ratio, droplet size, distribution of the drug in the phase system and the rate of diffusion. As can be seen from Figure 5, microemulsions provided the release of a lower amount of drug over 24 h with respect to the control. This result agreed with previously reported data [51,52] stating that microemulsions had lower release rates than solutions due to the presence of the tight packing of the surfactant layer, which retards the release of the drug from the oil phase. Among all of the formulations, M3 showed the fastest drug release rate (*p* < 0.05), which can be attributed to its viscosity characteristics. In fact, M3, bearing the lowest viscosity, provided the release of a greater cumulative amount of the drug after 24 h with respect to M1 and M2 (*p* < 0.05). As a matter of fact, it is known that a lower viscosity of a formulation allows for an easier drug diffusion through the vehicle than a higher viscosity and, consequently, a greater drug release [37]. Additionally, despite showing a greater viscosity than M1, after 24 h, M2 provided the release of a greater amount of AZT (*p* < 0.05). This result could be attributed to the lower size of M2 droplets than M1, which ensured a larger surface area for a more effective drug delivery [39]. However, in all of the cases, the prolonged release could be favorable for achieving suitable local drug concentrations over time and obtaining a more prolonged effect, thus minimizing the dosing and improving the effectiveness of skin infection treatment [53,54].

### 2.7. In Vitro Skin Penetration/Retention Studies

Skin penetration and retention studies were conducted in order to determine the AZT amount permeated and retained within the skin. This kind of evaluation represents a crucial aspect concerning the topical antibiotic application since it allows us to determine the possibility of reaching a sufficient local drug concentration, which is generally required to improve the treatment of skin infections. Moreover, this study was carried out by employing porcine ear full-thickness skin as a model, considering that the trends observed for the penetration of drugs into the porcine skin are highly representative of the in vivo situation on the human one [55].

The results collected from these experiments highlighted that, 6 h after the application of the samples in the donor compartment, only a small percentage of AZT was found to be able to diffuse through the skin and reach the receptor chamber. In particular, the cumulative percentage amount measured inside the receptor compartment was equal to 1.64 ± 0.69%, 1.25 ± 0.35% and 2.14 ± 0.85% for M1, M2 and M3, respectively (no significant difference was observed between the different microemulsions; *p* > 0.05). On the contrary, the AZT solution (control) penetrated the skin to a greater extent with respect to the developed microemulsions, since a significantly higher cumulative drug percentage was measured (8.79 ± 0.77%; *p* < 0.05).

**Figure 6:**
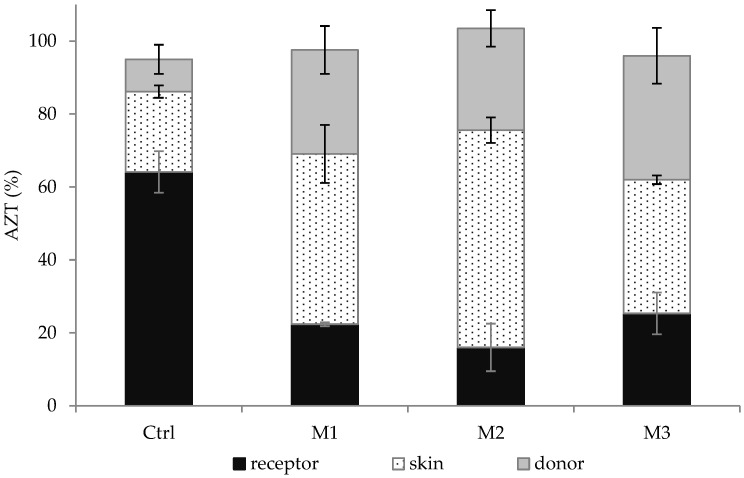
Percentage amount of AZT in the receptor compartment, within the skin and in the donor compartment obtained after 24 h from the application of AZT solution (control, Ctrl) or microemulsions. Data are expressed as means ± SD, n = 5.

The trend observed after 6 h was also confirmed by data obtained at the end of the experiment. Figure 6 shows the cumulative percentage amount of AZT in the donor chamber, the skin and the receptor compartment after 24 h. As can be seen, a more evident accumulation of the drug inside the receptor chamber was obtained when the control was used as a sample with respect to microemulsions. Specifically, the cumulative percentage amounts measured for the control, M1, M2 and M3 were equal to 64.13 ± 5.69%, 22.37 ± 0.55%, 16.00 ± 6.51% and 25.33 ± 5.73%, respectively (no significant difference between the different microemulsions was observed; *p* > 0.05). This result could be attributed to the different release behavior of the microemulsions and the control (as reported in the previous section), and to a different partition of the drug between the vehicle placed in the donor chamber, the skin and the receptor compartment. These findings clearly indicated that microemulsions could reduce undesired AZT penetration through the skin, which represents a fundamental point for the treatment of topical skin infections, also considering the need for lowering systemic AZT absorption.

Furthermore, a different retention of AZT within the skin was also evident. Indeed, among all of the samples, the lowest percentage of AZT retained inside the skin was obtained in the presence of the control (22.04 ± 3.99%), whereas AZT accumulated into the skin in considerable and greater amounts after the application of the different microemulsions (*p* < 0.05). This result might be attributed to the following reasons: (1) composition, droplet size and zeta potential of the microemulsions, (2) diffusion and retention of free AZT or AZT-loaded microemulsions through and within the skin. In particular, it is well-known that microemulsions can enhance the drug retention in the skin due to the virtue of their solubilizing potential, allowing for the partition of the dissolved drug into the skin and their interaction with skin lipids, in addition to their nanometric size [56]. Furthermore, as previously described, the negative zeta potential of microemulsions could also contribute to enhancing drug accumulation inside the skin [56].

Interestingly, the formulations play a certain role in determining the retention of AZT within the skin, and differences were observed between the developed microemulsions. In fact, 24 h after the sample application, the cumulative percentage amounts inside the skin were 46.69 ± 7.96%, 59.58 ± 3.49% and 36.65 ± 1.19% for M1, M2 and M3, respectively. M3, containing the lowest amount of Transcutol^®^ P, provided the lowest drug retention within the skin. It has been reported that Transcutol^®^ P is able to increase the skin accumulation of drugs by creating an “intracutaneous depot” [57]. This effect is probably created by the swelling of stratum corneum intercellular lipids without alteration of their multiple bilayer structure, in which, lipophilic compounds can be retained [58,59,60]. Moreover, although M2 contained a lower amount of Transcutol^®^ P with respect to M1, M2 promoted AZT accumulation within the skin to a greater extent than M1 (*p* < 0.05). This is probably influenced by the M2 droplet size, in agreement with previous findings that established that smaller droplets can greatly interact with the stratum corneum, which, in turn, improves the efficiency of skin retention [40].

To conclude, we can assume that all of the developed microemulsions, especially M1 and M2, can be considered as promising nanocarriers for the treatment of skin infections thanks to their ability to promote AZT retention inside the skin. This result is crucial in order to reach the final objective of improving the treatment of skin infections. In fact, owing to a lack of retention of most antibiotics into the skin from conventional topical formulations, skin infections generally do not respond to topical therapy with antibiotics. The possibility of reaching an adequate amount of the drug inside the skin through the topical application of the developed AZT-loaded microemulsions can (1) reduce the administration frequency, thus increasing patient compliance and (2) limit the risk of drug overuse, which is generally responsible for antimicrobial resistance. Both aspects can finally contribute to obtaining an advanced and improved treatment of bacterial skin infections.

### 2.8. Antimicrobial Activity

The antimicrobial activity of free AZT and AZT-loaded microemulsions was assessed against the *S. aureus* reference strain (ATCC 29213) and three clinical isolates displaying different antibiotic resistance profiles (strains #7 and #83 are MRSA, strain #88 is resistant to aminoglycosides) [61]. AZT MIC values are reported in Table 3. *S. aureus* ATCC 29213 displayed a MIC of 1 µg/mL, in accordance with values reported by EUCAST in MIC determination quality control guidelines [62]. *S. aureus* clinical isolates resulted in being slightly less susceptible to AZT, with MIC values of 2 µg/mL (strains #83 and #88) or 4 µg/mL (strain #7). For loaded microemulsions, MIC values in the range of 1–4 µg/mL were also registered. The inclusion of AZT in M1 led to a MIC equal to that of free AZT in three strains out of four (except for strain #88, two-fold MIC). M2 displayed a MIC equal to that of free AZT for strains #7 and #83 and two-fold MIC for strains ATTC 29213 and #88. M3 displayed a MIC equal to that of free AZT for strains #7 and two-fold MIC for strains ATTC 29213, #83 and #88. Unloaded microemulsions did not display antibacterial activity in the tested concentration range (data not shown). On the basis of antimicrobial tests, M1 showed the best activity profile, retaining an AZT efficacy on *S. aureus* reference strain ATTC 29213 and the tested MRSA (strains #7 and #83). The present microbiological data demonstrated that M1 can be proposed as an innovative formulation for the treatment of skin infections caused by *S. aureus*, as well as by MRSA.

### 2.9. Biocompatibility Studies

**Figure 7:**
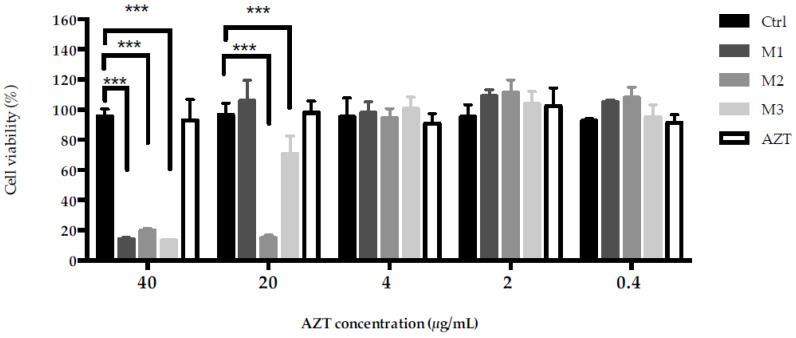
Fibroblasts viability (%) after 24 h of incubation at 37 °C with loaded microemulsions and AZT solution at different drug concentrations. The values denote the mean ± S.D. (n = 4). All treatments were analyzed against control (Ctrl), *** *p* < 0.001.

In order to assess the biocompatibility of loaded microemulsions, the viability of WS1 fibroblasts was analyzed by an MTT assay. Moreover, it was investigated if the WS1 cells’ viability was affected by AZT. Fibroblasts were treated with loaded microemulsions and an AZT solution at final drug concentrations ranging from 0.4 to 40 µg/mL, which covered and exceeded the MIC values previously observed. The results, reported in Figure 7, showed the absence of a toxic effect after treatment with AZT under all of the tested concentrations, as a viable population is superimposable to the control ones. All of the loaded microemulsions exerted toxicity when diluted at a final AZT concentration of 40 µg/mL. At an AZT concentration of 20 µg/mL, M1 showed a superior biocompatibility with respect to M2 and M3, even if, for M3, the cell viability was around 70%, which is considered to be the non-cytotoxic threshold [63]. The lowest biocompatibility registered for M2 could be attributed to its highest surfactant content among all of the formulations. However, it can be highlighted that all of the loaded microemulsions are highly biocompatible when diluted at a final AZT concentration equal to 4 µg/mL, which is the highest measured MIC value. The same trend was observed for unloaded microemulsions (data not shown). The obtained data revealed that all loaded microemulsions were not cytotoxic under the highest MIC of AZT and that, notably, M1 was the formulation with the best biocompatibility profile. Considering these results, M1 could be considered safe for skin application, even if further in vivo studies should be performed to confirm this hypothesis.

## 3. Materials and Methods

### 3.1. Materials

Castor oil, sunflower oil and vitamin E acetate (tocopherol acetate, 96%) were obtained from ACEF (Piacenza, Italy). Labrafac^®^ CC (medium chain triglycerides), Labrafil^®^ M 2125 CS (linoleoyl polyoxyl-6-glycerides), Labrasol^®^ (caprylocaproyl polyoxyl-8 glicerides), Capryol^®^ 90 (propylene glycol monocaprylate) and Transcutol^®^ P (diethylene glycol monoethyl ether) were received as a generous gift from Gattefossé (Lyon, France). Tween 80 (polyoxyethylene sorbitan mono-oleate) and azithromycin dihydrate (AZT) were provided by Fluka (Milan, Italy) and Merck (Milan, Italy), respectively. Propylene glycol was purchased from Carlo Erba (Milan, Italy). All other chemicals of analytical grade were from Sigma-Aldrich (Milan, Italy). For in vitro release and permeation tests, a buffer solution at pH 7.4 (PBS) based on 7.4 mM Na_2_HPO_4_∙10H_2_O, 1.1 mM KH_2_PO_4_ and 136 mM NaCl was used. A MilliQ apparatus by Millipore (Milford, MA, USA) was used to obtain ultrapure water (18.2 MΩ cm).

### 3.2. Solubility of AZT

The solubility of AZT in different oils, surfactants and co-surfactants was estimated by dispersing an excess of AZT in 1 mL of each of the selected components. Suspensions were vortexed and then kept at room temperature under stirring (200 rpm) for 72 h. The samples were centrifuged at 14,500 rpm for 20 min (Microspin 12, Highspeed Mini-centrifuge, Biosan, Riga, Latvia) to remove the excess of AZT. The supernatants were subsequently diluted in methanol and analyzed through HPLC assay (Section 3.3) to determine the amount of dissolved AZT.

### 3.3. HPLC Assay for AZT

Quantification of AZT was performed by HPLC. The chromatographic system was composed of a Shimadzu (Milan, Italy) LC-10ATVP chromatographic pump and a Shimadzu SPD-10AVP UV-vis detector set at 215 nm. Separation was obtained on a Phenomenex (Torrance, CA, USA) Kinetex (150 mm × 4.6 mm I.D., 5 mm) coupled to a Phenomenex (Torrance, CA, USA) Security Guard C18 guard cartridge (4 mm x 3.0 mm I.D., 5 mm). A phosphate-buffered saline was prepared by dissolving KH_2_PO_4_ in purified water (0.01 M), and the pH was adjusted to 7.5 by adding 10M KOH. The buffer was then filtered through cellulose nitrate membrane filters (0.45 μm pore size; Sartorius AG, Göttingen, Germany). The mobile phase was composed of a mixture of phosphate-buffered saline at pH 7.5, methanol and acetonitrile (10/50/40, *v*/*v*). The flow rate was 0.8 mL/min. Solutions of AZT in methanol at drug concentrations ranging from 13 μg/mL to 400 μg/mL were used to construct a standard curve (R^2^ = 0.9964). For the evaluation of drug diffused through the skin, another calibration curve of AZT in PBS pH 7.4/ ethanol (80:20 *v*/*v*) was obtained, with drug concentrations ranging from 0.5 μg/mL to 50 μg/mL (R^2^ = 0.9943).

### 3.4. Pseudo Ternary Phase Diagrams

The excipients used for microemulsion preparation were selected in relation to their solubilizing potential for AZT. The selected components were vitamin E acetate (oil phase), Labrasol^®^ (surfactant), Transcutol^®^ P (co-surfactant) and ultrapure water (aqueous phase). Pseudo ternary phase diagrams were constructed to investigate the concentration range of components for microemulsion formation. They were built through the water titration method using oil, a mixture (S_mix_) of surfactant and cosurfactant and water. The mixture of surfactant and co-surfactant (S_mix_) was separately prepared by weighing accurate amounts of Labrasol^®^ and Transcutol^®^ P in order to obtain 1:1, 2:1 and 3:1 (*v*/*v*) ratios of surfactant and cosurfactant (density values of Labrasol^®^ and Transcutol^®^ P equal to 1.06 g/mL and 0.99 g/mL, respectively); the mixture was then vortexed vigorously for 30 s. For fixed ratios of oil/S_mix_ (1/9, 1.25/8.75, 2/8, 3/7, 4/6, 5/5, 6/4, 7/3, 8/2, 9/1 *v*/*v*), increasing amounts of ultrapure water of between 5 and 95% *w*/*w* of total emulsion content were added dropwise under stirring (200 rpm). After each addition, the mixtures were stirred for 15 min, left 5 min to rest and finally visually examined for transparency or appearance of turbidity. Using this information, pseudo-ternary phase diagrams were plotted and the optimized formula for microemulsions was selected.

### 3.5. Selection and Preparation of Microemulsions

#### 3.5.1. Preparation of Unloaded Microemulsions

Suitable compositions were identified from the pseudo-ternary phase diagram to develop final formulations (Table 1). Precisely, microemulsions with the following oil:S_mix_:water (*w*/*w*/*w*) ratios were selected for further experiments: 7.7:58.8:33.5 for M1 (S_mix_ 2:1 *v*/*v*), 7.7:59.1:33.2 for M2 (S_mix_ 3:1 *v*/*v*) and 5.9:45.3:48.8 for M3 (S_mix_ 3:1 *v*/*v*). Microemulsions were prepared by mixing the different components into glass vials under magnetic stirring as previously described (Section 3.4). Finally, to establish the type of microemulsion [32,64], electrical conductivity was measured at +25 °C using a conductometer GLP 31 (CRISON 2000, Modena, Italy).

#### 3.5.2. Content Determination and Preparation of Loaded Microemulsions

In order to determine the maximum loading content of AZT in the selected microemulsions, an excess amount of drug was placed into the oil phase containing S_mix_, after which, water was added. It was further maintained under stirring at 200 rpm for 48 h at +25 °C. Excess AZT was removed by centrifugation at 14,500 rpm for 20 min, the supernatant was diluted with methanol and the content of AZT dissolved in the microemulsions was measured by HPLC. For the preparation of AZT-loaded microemulsions, AZT was dissolved into the mixture composed of vitamin E acetate and S_mix_ and then water was added dropwise (conditions reported in Section 3.4), obtaining a final drug concentration of 10 mg/mL.

### 3.6. Characterization of Microemulsions

#### 3.6.1. Determination of pH and Viscosity

The pH of the selected microemulsions was determined through a pH meter (MicroPH CRISON 2000, Modena, Italy). The viscosity was measured with a falling ball viscometer at +25 °C (HAAKETM Falling Ball Viscometer Type C, Thermo electron corporation, Karlsruhe, Germany).

#### 3.6.2. Mean Droplet Size, Size Distribution and Zeta Potential Measurements

The droplet size and distribution (PDI) of the selected microemulsions were measured at +25 °C by photon correlation spectroscopy (PCS) using a Brookhaven 90-PLUS instrument (Brookhaven Instruments Corp., Holtsville, NY, USA) with a He-Ne laser beam at a wavelength of 532 nm (scattering angle of 90°). Zeta potential measurements were carried out at +25 °C on a Malvern Zetasizer 3000 HS instrument (Malvern Panalytical Ltd., Malvern, UK).

### 3.7. Stability

To evaluate the thermodynamic stability of the microemulsions, different tests, including centrifugation and freeze–thaw cycle methods, were performed [65,66,67]. Briefly, the selected formulations were centrifuged at 3500 rpm for 30 min and three cycles, between freeze temperature (−20 °C) and room temperature +25 °C with storage at each temperature for no less than 24 h, were conducted. The phase separation, transparency and/or drug precipitation from formulations were evaluated. Moreover, mean droplet size and PDI of the microemulsions were monitored over a period of storage of 180 days at +4–8 °C and +25 °C. At determined time intervals, changes in vesicle size and PDI were monitored using PCS.

### 3.8. In Vitro Release Studies

The release of AZT from the selected microemulsions was determined on a Franz cell diffusion system under sink conditions using a dialysis membrane (Mw cut off 12–14,000 Da; VWR International, Milan, Italy) and a heating circulator set to +32 °C [14]. A Franz-type static glass diffusion cell (15 mm jacketed cell with a flat ground joint and clear glass with a 12 mL receptor volume; diffusion surface area = 1.77 cm^2^), equipped with a V6A Stirrer (PermeGearInc., Hellertown, PA, USA) was employed. The formulation (0.2 mL) was placed in the donor chamber, whereas the receiver chamber was filled with a mixture of PBS and ethanol (80:20, *v*/*v*) and mixed with a magnetic stirrer. At predetermined time intervals until 24 h, samples (0.2 mL) were collected from the receiver chamber, replaced with the same amount of fresh medium and analyzed using HPLC. A control solution containing an equivalent amount of AZT dissolved in a mixture of ethanol and water (60:40, *v*/*v*) was also tested. The results of in vitro release studies are shown as cumulative drug amount released (expressed as fractional amount Mt/M0, where Mt represents the amount of AZT released at each time and M0 the total AZT mass loaded into microemulsions) plotted as a function of time.

### 3.9. In Vitro Skin Penetration/Retention Studies

A porcine ear skin model was used to assess the skin penetration and retention of AZT from the different microemulsions and control. Porcine ear skin was used as model due to its similarity to the human one in terms of morphology and permeation characteristics [68,69]. Freshly excised pig ear was provided from a local abattoir (CLAI, Faenza, Italy) and washed with saline solution. Then, full-thickness skin was obtained by carefully isolating the cartilage and excising the subcutaneous tissue [70]; finally, it was frozen at −20 °C on aluminum foil and used within 1 month. The integrity of the full-thickness skin was evaluated by measuring the electrical resistance (voltage: 100 mV, frequency: 100 Hz; Agilent 4263B LCR Meter, Microlease, I). Membrane samples with an electrical resistance below 1.57 kΩ/cm^2^ were excluded from permeation experiments, in agreement with electrical resistance values recommended in literature [71,72].

Before the experiments, the skin was defrosted for 15 min and placed between the donor and receiver chambers of the Franz-cell with the stratum corneum facing the donor compartment. The receptor compartment was composed of 12 mL of PBS and ethanol (80:20, *v*/*v*), maintained at 32 °C by means of a surrounding jacket and constantly stirred to assure a uniform drug concentration. A total of 0.2 mL of the formulation or control (containing an equivalent amount of AZT dissolved in a mixture of ethanol and water; 60:40, *v*/*v*) was placed in the donor chamber. At predetermined time intervals until 24 h, samples (0.2 mL) were collected from the receiver compartment, replaced with the same amount of fresh medium and analyzed using HPLC. At the end of the experiment, the liquid of the donor compartment was removed and the skin surface was carefully rinsed with methanol (0.5 mL). Both fractions were diluted in methanol and analyzed through HPLC in order to determine the non-penetrated drug (AZT in the donor compartment). Subsequently, the skin was dismounted from the cells, cut into very small pieces and placed in methanol (5 mL) for 5 h under magnetic stirrer (300 rpm) to extract the drug. Finally, skin was removed and the solution was centrifuged at 14,500 rpm for 20 min and analyzed through HPLC to determine AZT amount retained within the skin. The results of in vitro penetration/retention studies are shown as percentage of drug amount inside the donor compartment, the skin and the receptor chamber.

### 3.10. Antimicrobial Activity

The antimicrobial activity of AZT and microemulsions was tested against *S. aureus* ATCC 29213 and three clinical multi-drug-resistant *S. aureus* isolates, namely strain #7 (resistant to beta-lactams, fluoroquinolones, ansamycins), strain #83 (resistant to beta-lactams, fluoroquinolones) and strain #88 (resistant to aminoglycosides) [59]. *S. aureus* strains were routinely grown in nutrient broth (NB), at 37 °C. A two-fold microdilution assay was performed on a 96-well plate, following NCCLS standard guidelines [73], and minimal inhibitory concentrations (MIC) were determined. Loaded microemulsions and AZT solution (containing an equivalent amount of AZT dissolved in a mixture of ethanol and water; 60:40, *v*/*v*) were diluted in NB to obtain an AZT concentration of 62.5 µg/mL; then, they were tested in the range of 0.24–31.25 µg/mL AZT. Unloaded microemulsions were diluted and tested under the same conditions to assess possible antibacterial effect of microemulsion themselves. MIC values were determined after 24 h of incubation at 37 °C.

### 3.11. Biocompatibility Studies

Biocompatibility was tested by MTT assay on WS1, a human fibroblast cell line (American Type Culture Collection, ATCC, Manassas, VA, USA). Cells were seeded at 5000 cells per well in a 96-well plate (Corning^®^, NY, USA) in DMEM supplemented with D-glucose (4.5 g/L), 10% FBS (*v*/*v*) and 2 mM L-glutamine (Euroclone S.p.A., Milan, Italy). After incubation in 5% CO_2_/ humidified air at 37 °C for 24 h, cells were treated with microemulsions and AZT solution (containing an equivalent amount of AZT dissolved in a mixture of ethanol and water; 60:40, *v*/*v*) at increasing drug concentrations (0.4, 2, 4, 20 and 40 µg/mL). At 24 h of treatment, 10µL of 3-(4,5-Dimethyl-2-thiazolyl)-2,5-diphenyl-2*H*-tetrazolium bromide (MTT) (5 mg/mL stock solution) (Merk KGaA, Darmstadt, Germany) was added to each well and the plate was incubated for 4 h. After 4 h, the cell culture medium was removed and the formazan crystals were solubilized by adding 100 µL/well of propan-2-ol (Merk KGaA, Darmstadt, Germany). The absorbance at 570 nm was measured and recorded with a plate reader (TECAN, Männedorf, Switzerland). The sample absorbance at 690 nm was used as reference wavelength for correction.

### 3.12. Statistical Analysis

All experiments were carried out in triplicate, while in vitro skin penetration/deposition and biocompatibility studies were carried out with five and four replicas, respectively. Results are expressed as mean ± SD. T-test was used to determine statistical significance of studies. For biocompatibility results, two-way ANOVA was performed with GraphPad Prism. The criterion for statistical significance was *p* < 0.05.

## 4. Conclusions

The topical treatment of skin infections remains a challenge, mainly due to limited drug retention inside the skin and the growing emergence of antibiotic-resistant strains of *S. aureus*. The design of appropriate nanocarriers loaded with antimicrobial drugs appears as a great opportunity to improve conventional therapies. In this context, our study proposed AZT-loaded microemulsions as a promising and innovative tool for the treatment of bacterial skin infections. The development of microemulsions based on vitamin E acetate, Labrasol^®^ and Transcutol^®^ P allowed us to improve AZT solubilization. Moreover, physico-chemical characterization evidenced that the microemulsion composition influenced parameters, such as the viscosity and the droplet size. The obtained findings also demonstrated for the first time that AZT-loaded microemulsions were able to guarantee a prolonged drug release and a greater drug accumulation inside the porcine skin with respect to the control. The latter properties could ensure minimizing the dosing and the administration frequency and limiting the risk of drug overuse, which is generally responsible for antimicrobial resistance, thus finally improving the effectiveness of skin infection treatment. Additionally, loaded microemulsions retained antibacterial activity; notably, M1 was as effective as free AZT against the *S. aureus* reference strain and MRSA, and was characterized by the best biocompatibility profile. To conclude, the present study can open new avenues for the treatment of skin infections caused by *S. aureus* and MRSA through the topical application of AZT-loaded microemulsions as an alternative to conventional formulations. In addition to these promising findings, some advantages of microemulsions, such as their stability and the ease of manufacturing, make them easily scalable without particular equipment.

Future studies will be performed in order to deeply characterize the morphological properties of the microemulsions and to evaluate their efficacy on animal models.

## Figures and Tables

**Table 1:** Components (% *w*/*w*) of the selected microemulsions.

Components	M1	M2	M3
Vitamin E acetate	7.7	7.7	5.9
Labrasol^®^	40.1	45.1	34.6
Transcutol^®^ P	18.7	14.0	10.7
Water	33.5	33.2	48.8

**Table 2:** Size (nm), PDI and zeta potential (mV) of the selected microemulsions.

Formulation	Size (nm)		PDI		Zeta Potential (mV)	
	Unloaded	Loaded	Unloaded	Loaded	Unloaded	Loaded
M1	120.8 ± 5.4	138.1 ± 1.3	0.188 ± 0.069	0.279 ± 0.018	−32.60 ± 1.71	−26.87 ± 0.32
M2	93.8 ± 1.1	97.4 ± 1.2	0.170 ± 0.038	0.189 ± 0.071	−31.76 ± 0.82	−27.78 ± 1.28
M3	106.6 ± 7.1	129.8 ± 0.6	0.195 ± 0.032	0.247 ± 0.004	−27.10 ±1.21	−22.32 ± 0.89

**Table 3:** MIC (µg/mL) of free AZT and loaded microemulsions against *S. aureus* strains.

*S. aureus* Strain	AZT	M1	M2	M3
ATCC 29213	1	1	2	2
#7	4	4	4	4
#83	2	2	2	4
#88	2	4	4	4

## Data Availability

Data are contained within the article.
